# Imaging Features of Primary T Cell Lymphoma in Bone: A Case Report and Review of Literature

**DOI:** 10.3389/fonc.2021.690819

**Published:** 2021-08-09

**Authors:** Suli Yu, Jianqiang Xu

**Affiliations:** ^1^Department of Hand and Upper Extremity Surgery, Jing’an District Central Hospital, Shanghai, China; ^2^Department of Orthopedics, Ruijin Hospital, Shanghai Jiao Tong University School of Medicine, Shanghai, China

**Keywords:** case report, primary bone lymphoma, peripheral T cell lymphoma, imaging feature, treatment strategy

## Abstract

Primary bone lymphoma (PBL) is a less frequent type of extranodal lymphoma, which is defined as a single skeletal tumor or multiple bone lesions without visceral or lymph node involvement. Most published cases have reported diffused large B cell lymphoma (DLBCL) of PBL, and the prognosis is good after conventional treatment. Primary T-cell lymphoma is extremely rare in the literature. The clinical symptoms, imaging findings, diagnosis, treatment and prognosis of primary T-cell lymphoma of bone are still unclear. The case details a young male patient who was treated for bone tuberculosis and was diagnosed with T-cell lymphoma during an open surgical biopsy. Further imaging evidence showed the lymphoma was localized within the femur. The patient responded poorly to combined chemo- and radiotherapy. He was confirmed with local lung metastases 11 months later and died at the 17th month of onset. I would like to provide PBL entities with some rare information about primary bone peripheral T-cell lymphoma and discuss the best strategy for the treatment of rare PBL subtypes.

## Introduction

Lymphomas commonly occur in the lymph nodes, and extranodal sites are less frequent origins of lymphomas. Primary bone lymphoma (PBL) accounts for 4–5% of extranodal non-Hodgkin’s lymphomas and less than 1% of all malignant lymphomas, and 7% of malignant primary bone tumors (1). Primary bone lymphoma was first described by Oberling in 1928 (2). Parker and Jackson reported 17 cases in 1939 (3) under the designation reticulum cell sarcoma of bone, establishing PBL as a distinct clinical entity. It must be distinguished from the more common condition characterized by secondary bone involvement of systemic lymphomas. More than 20% of lymphoma patients have secondary bone involvement (4).

According to the revised European and American lymphoma (REAL) classification (5), most PBLs are diffuse large B-cell lymphoma (DLBCL). Primary T cell lymphoma is extremely rare in the literature. Especially the management of T-cell lymphoma has been largely extrapolated from the treatment of aggressive B-cell lymphomas and they possess a poorer prognosis (6). Since other subtypes of PBL are less common, there is little literature that provided sufficient evidence for clinical outcomes and therapeutic management. Here, the study demonstrated the clinical evolution of a peripheral T cell lymphoma case with good prognostic factors, but responded poorly to the standard and recommended therapy.

## Case Presentation

**Figure 1:**
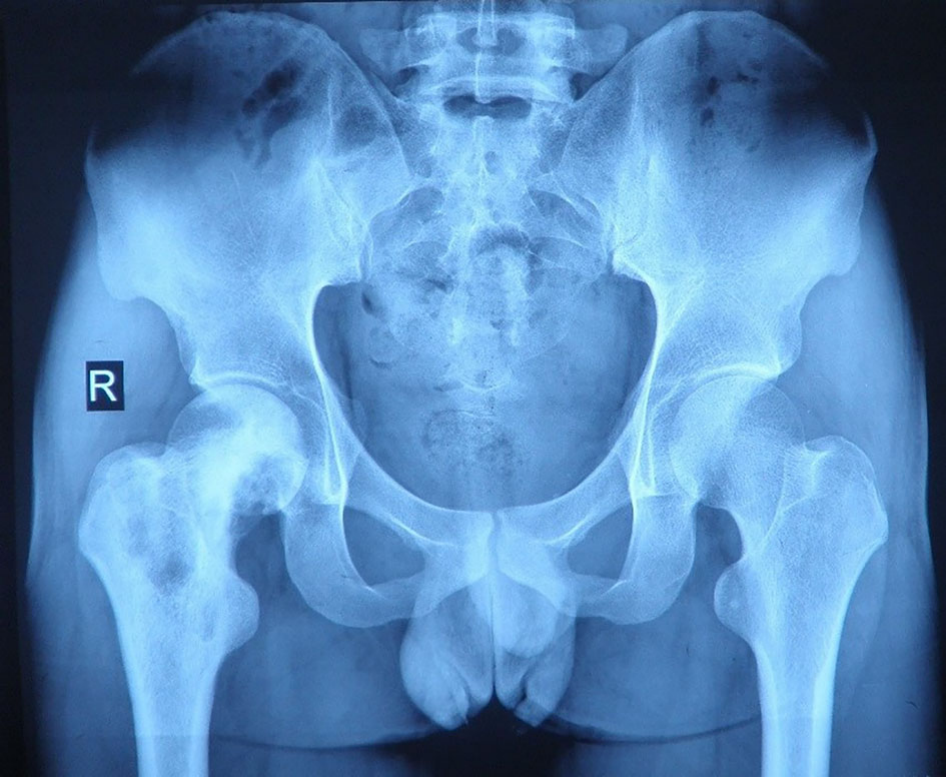
Plain radiography of pelvis at the first week of local pain showed flake bone destruction in right femoral trochanter.

**Figure 2:**
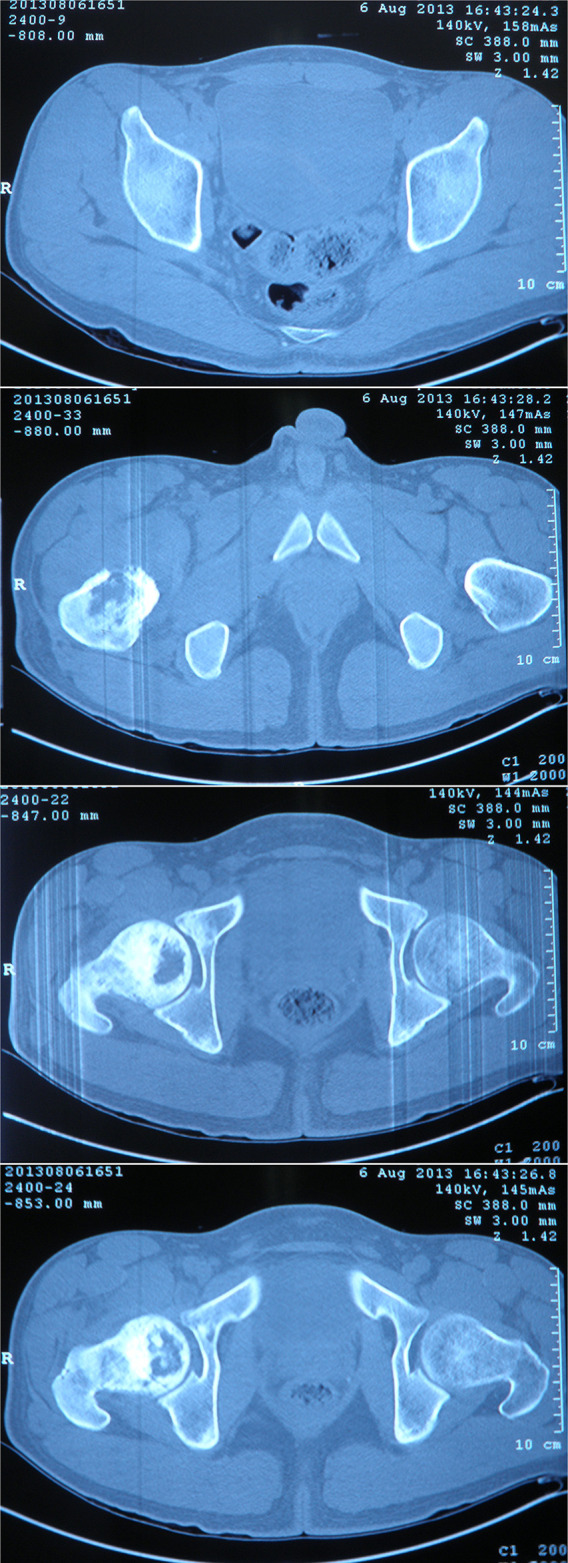
CT scan of pelvis at the first week of onset demonstrated a permeative pattern of bone destruction in the femerol head and neck which poorly marginated with laminar periosteal reaction. A high bone density lesion was detected under the articular surface of right acetabulum. The breadth of the joint space was normal.

**Figure 3:**
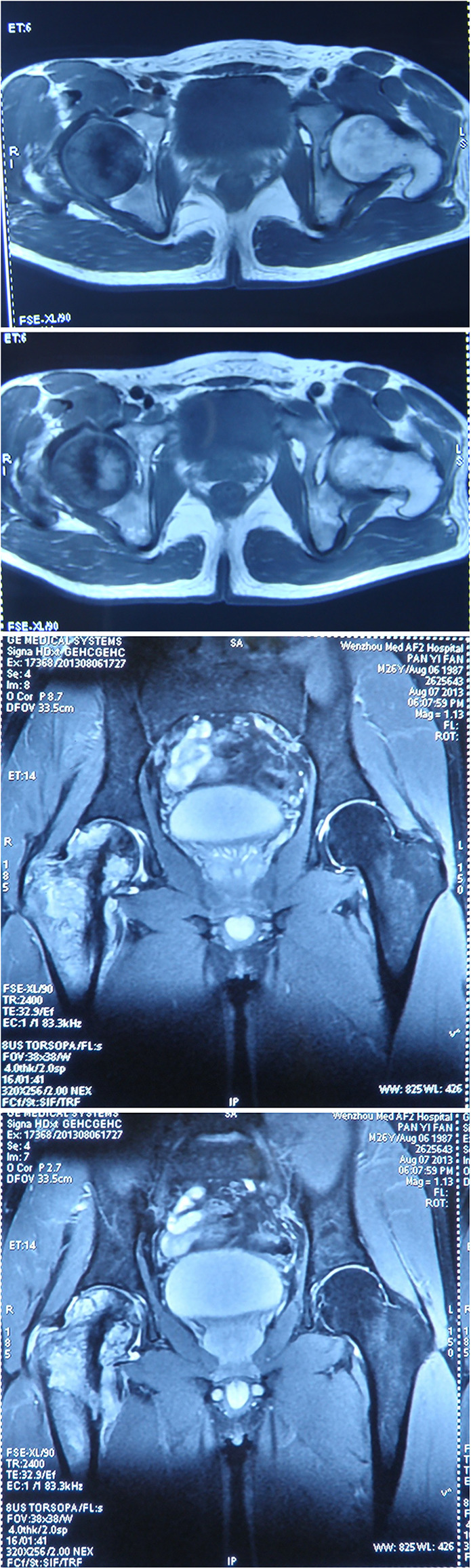
MRI of pelvis at the first week of onset presented a wide range capsule-patterned bone destruction of right femoral head and trochanter with high signal intensity in T2-weight imaging (T2WI) while lower and mixed signal intensity in T1-weight imaging (T1WI). Cortical breakthrough was detected on the anterior of femoral trochanter, and surrounding soft tissue was swelling while acetabulum and joint space was normal.

A 26-year-old Chinese male with a firm palpable swelling mass (maximum size of 9.6 cm × 7.8 cm) around the right hip came to my hospital for medical help. Six months ago, he started a gradual onset of pain in the upper right femur without obvious cause. Plain radiography of pelvis (**Figure 1**) showed flake bone destruction in right femoral trochanter. To evaluate the lesion, computed tomography (CT) scan of the lesion (**Figure 2**) demonstrated poorly marginated bone destruction in the femerol head and neck. Moreover the magnetic resonance imaging (MRI) of pelvis (**Figure 3**) presented a wide range capsule-patterned bone destruction of right femoral head and trochanter.

Through a combined series of radiological examinations and patient’s clinic manifestations, tuberculosis was first considered due to its widespread in China. A T-SPOT test of the patient revealed a high value of 159.20 g/ml (reference range: 0–14 pg/ml). No lesions were found on the first lung CT scan, and the diagnosis of bone tuberculosis was made. Then antitubercular therapy with the rifampicin, isoniazid, and ethambutol was administrated. After half a month of treatment, the local pain in the right hip of the patient was not relieved, and the palpable mass gradually increased and new fever symptoms appeared. One month after antitubercular therapy, the full blood test revealed a slight increase in the percentage of neutrophils and eosinophils, and the ESR (Erythrocyte Sedimentation Rate) and CRP (C-reactive protein) values increased to 94 mm/h and 87.28 mg/l, respectively. A CT-guided needle biopsy of the lesion obtained an immunohistochemical report of CD68+ and CD163+, which indicated the presence of M2 macrophage (7). But the following application of antibiotics therapy did not relieve any symptoms.

**Figure 4:**
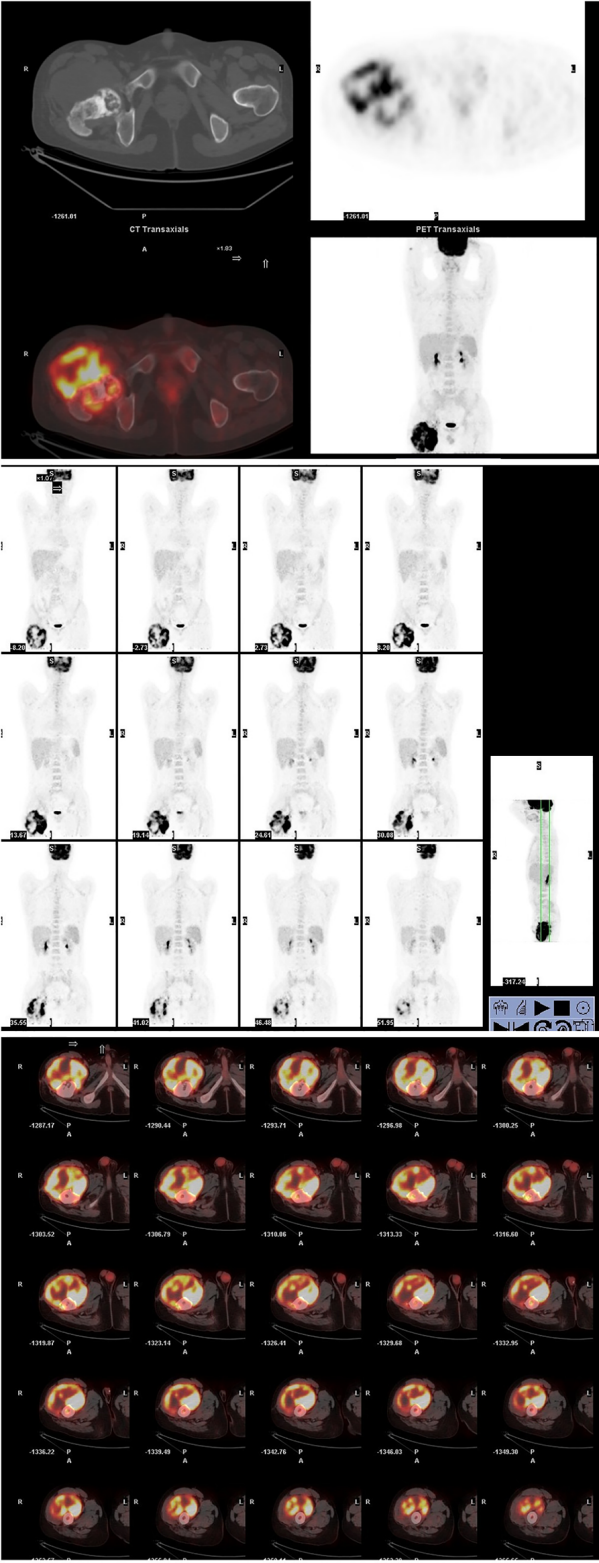
A whole-body tomography positron-emission tomography and computed tomography using ^18^F-fluorodeoxyglucose (^18^F-FDG) demonstrated a huge cauliflower-like appearance skeletal lesion with very high fluorodeoxyglucose uptake (SUV max 8.0) in the upper segment of right femur. It was noted that the ^18^F-FDG avidity was not detected in the lymph nodes or any internal organs.

**Figure 5:**
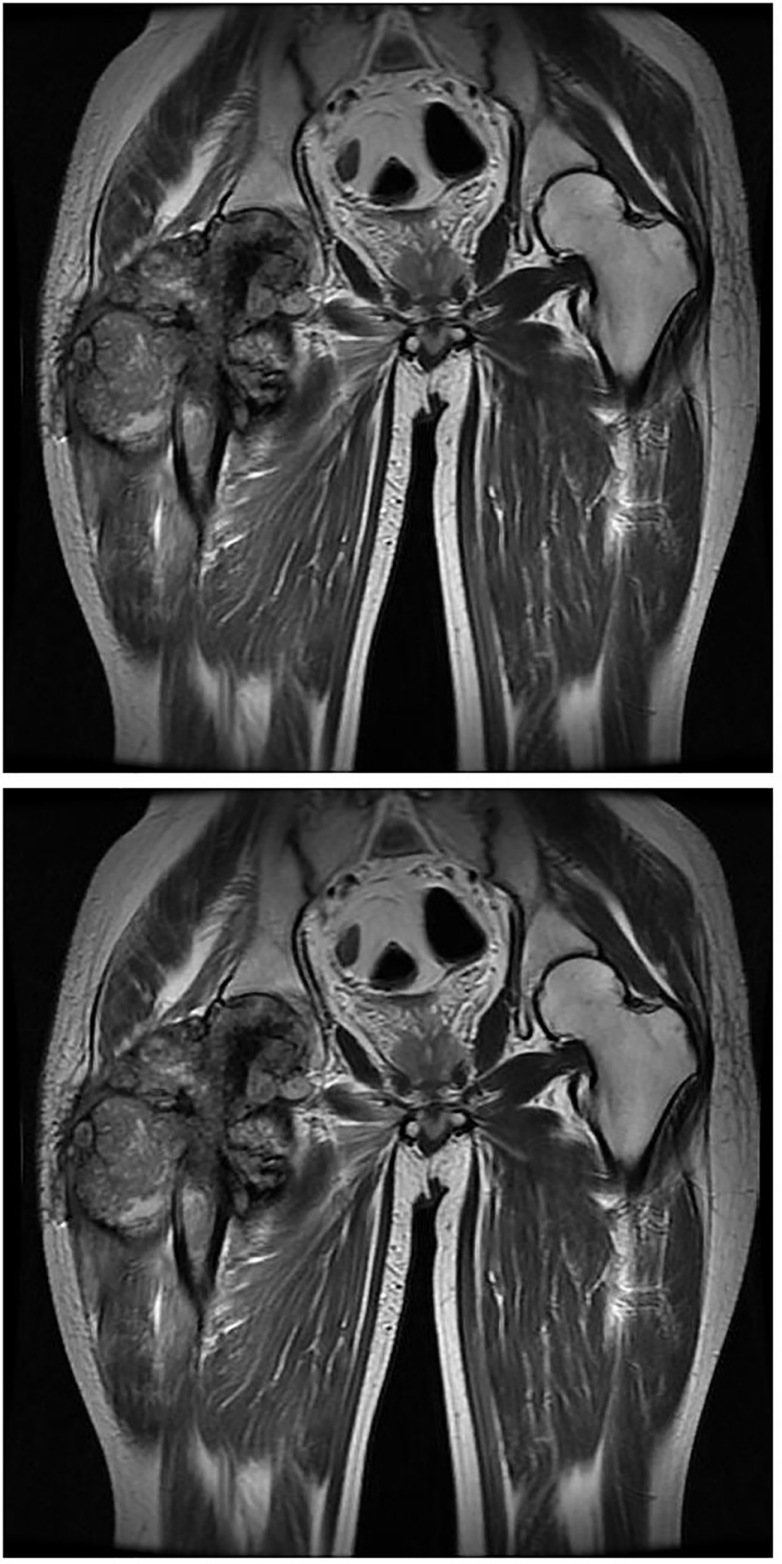
MRI imaging at the fourth month of onset detected bone destruction and low intensity in T1WI while mixed intensity in T2WI mainly localized at the upper segment of femur. It is surrounded by cauliflower-like soft tissue mass with size of approximate 12.6 cm by 21.5 cm in great dimension. Jacent gluteus, iliopsoas, musculi obturator extemus and muscle group of right thigh acetabulum were eroded.

**Figure 6:**
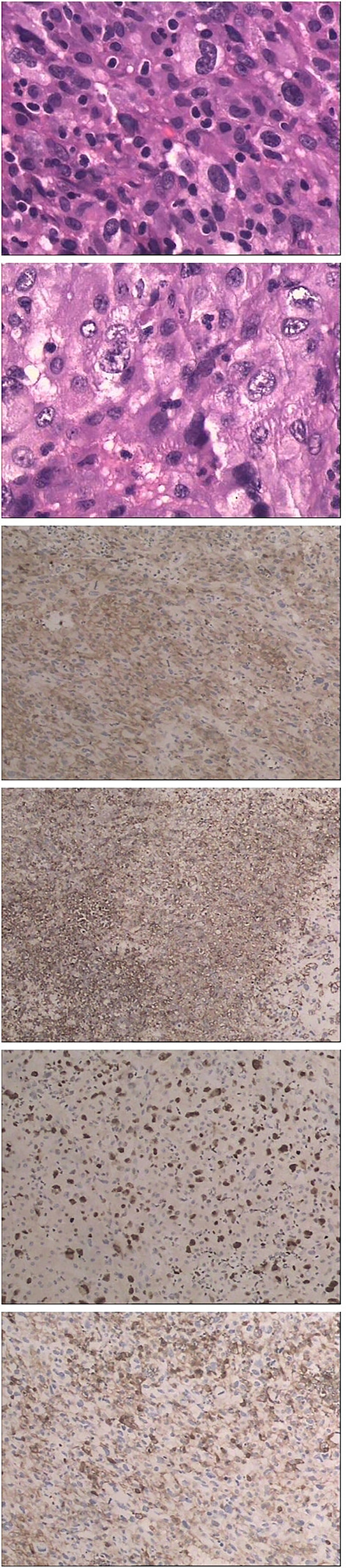
Hematoxylin and eosin (HE) and immunohistochemical staining shows CD4+, CD5-, CD43+, MIB-1 40%+ LCA+.

Out of suspicion of some malignant bone tumors, the patient was discontinued with the antitubercular drug administration and transmitted to the hospital. The whole-body positron-emission tomography and computed tomography (PET-CT) using ^18^F-fluorodeoxyglucose (^18^F-FDG) demonstrated a very high fluorodeoxyglucose uptake (SUV max 8.0) in the upper segment of the right femur (**Figure 4**) and magnetic resonance imaging (MRI) showed that a large cauliflower-like tumor tissue completely eroded the right articulation coxae (**Figure 5**). No similar lesions were found in the lymph nodes or any internal organs. In the following open surgery biopsy, the lesion tissue showed a gray and white fish-meat-like appearance, involving the articular cavity of the right hip joint. Variably shaped cells with abundant and pale cytoplasm were interspersed in a background of necrotic tissues upon histological examination. These cells presented prominent atypia (**Figure 6**). According to the REAL classification (5), these morphological and immunohistochemical features are consistent with peripheral T cell lymphoma-not otherwise specified (PTCL-NOS).

Since no involvement of lymphoid tumor cells was suggested in the staging of bone marrow biopsy, the final diagnosis was primary bone lymphoma, which was classified as stage-IBE (Ann-Arbor staging). The patient’s revised-International Prognostic Index (R-IPI) (8) score was 1 (the patient’s β2-microglobulin level and lactate dehydrogenase level remained normal reference) which indicated a good prognosis.

**Figure 7:**
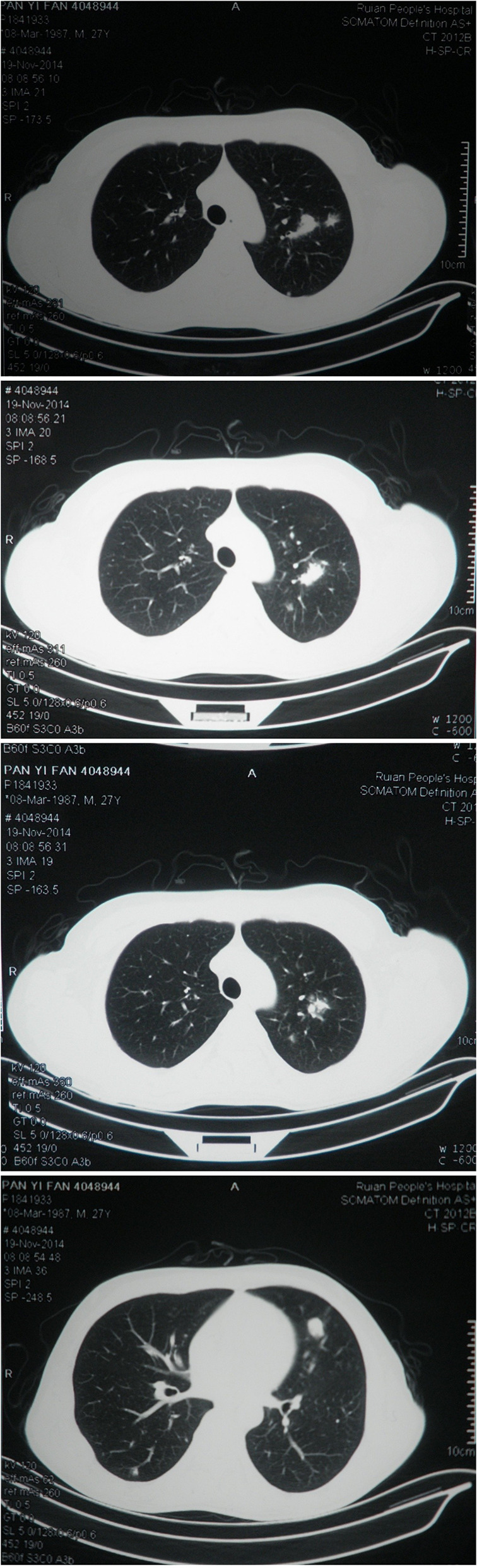
Pulmonary CT scan showed the multi-transfer lesions of lung at the 11th month of onset.

After four rounds of chemotherapy including a standard CHOP-like regimen, two cycles of ITE (Ifosfamide, Pirarubicin, and Etoposide), and an HD-MTX (high dose methotrexate) regimens, the regional metastatic lymph node was confirmed by biopsy at the 9th month of his onset, so two additional regimens including gemcitabine and cis-platinum were conducted. After chemotherapy, the tumor did not shrink significantly, and clinical symptoms such as fever, pain, and fatigue persisted. Due to his poor response to chemotherapy, radiotherapy was recommended. The patient received a total of 50 Gy of local radiotherapy and then claimed that the local pain and fever had ceased. But half a month later, the pain got worse. It was further recommended that patient adopt the treatment plan of amputation or bone marrow transplantation, and if the patient refuses resection treatment. While waiting for a bone marrow match, radiological evidence showed multiple metastatic lesions in his lungs at the 15th month of his onset (**Figure 7**), and the patient died 2 months later.

## Discussion

### History and Hypotheses of Pathogenesis

As a distinct clinical entity of extranodal lymphoma, the pathogenesis of PBL remains unclear even it was first described 90 years ago. Compared with the frequency of plasma cell tumors in bone, the occurrence of primary lymphoma of bone, even diffused large B cell lymphoma is the most common type, is relatively rare. Leval et al. (9) proposed that primary bone lymphoma may represent a tumor of post-germinal center B-cell bone lymphoma, because the bone marrow is the normal site of homing of plasma cells which functionally is defined as terminally-differentiated, immunoglobulin(Ig)-secreting post-germinal center B-cell (10).

Peripheral T-cell lymphomas (PTCL) are a group of rare lymphomas originating from mature (post-thymic or peripheral) T lymphocytes and NK cells (11), which comprise approximately 10–15% of all non-Hodgkin lymphomas (12–14) and represent more than 20% in Asian countries of all non-Hodgkin lymphoma (15–17). PTCL encompass a biologically and clinically heterogeneous group of rare neoplasia, and generally are aggressive tumors that carry a poor prognosis. For the lack of identification of molecular and genetic signature, 30–50% T cell lymphoma had been categorized in the not otherwise group (NOS). PTCL-NOS shows a propensity for extranodal involvement, where the most frequent extranodal sites of involvements are the gastrointestinal tract and the skin (18). But the sole bone invasion case had not been reported until date.

The diagnosis of PTCL is challenging due to the complexity of the histologic classification. The tumor sample quality must be high for PTCL diagnosis and a broad immunohistochemistry panel (19) is needed to establish. The false-positive T spot report may give a hint of the disarranged T cell lymphoma.

### Clinical and Radiology Performances

Patients with bone primary NHL have no typical symptoms, including insidious and intermittent local bone pain, soft tissue swelling, palpable masses or pathological fractures, and systemic symptoms such as fever and weight loss.

Research had showed a slight male preponderance, and most patients are over 45–50 years old (20). NHL of bone can arise in any part of the skeleton, the femur (29%) is the most common site of predilection, followed by: pelvis (19%), humerus (13%), skull (11%), and tibia (10%) (21).

The radiological findings are non-specific and are of little help in differentiating PBL from osteosarcoma, Ewing’s sarcoma, osteomyelitis, and metastatic disease. Therefore, the key role of histopathological immunophenotyping and immunohistochemical research on the diagnosis and confirmation of lymphoma cells is left to the biopsy samples. Needle biopsy is not adequate for pathological diagnosis since its limitation in handling biopsy tissue well. The tissue should be acquired without crush artifact or decalcification to preserve cell morphology.

### Treatment Method

Treatment of bone lymphoma has been varied during decades, but there are still controversies. At first, only local radiation was recommended to patients with single bone involvement. As to the development of lymphoma in molecular biology, chemotherapy was conducted on PBL patients and showed better outcomes, so radiotherapy was no longer used to the multifocal patients. In recent years, the concept of combined modality therapy has thrived. However, surgery is only indicated for pathologic fractures and biopsy to identify the pathology types of bone lesions. Bone marrow transplant is only used for a very small number of patients, and the outcome is still unclear.

Most studies have indicated that primary NHL of bone cases have favorable outcome compared with either secondary bone involvement lymphomas or other kinds of primary malignant bone tumors, especially when treated by combined modality therapy. Beal et al. (22) conducted a long-term follow-up of 82 PBL patients, and showed that the 5-year Overall survival (OS), cause-specific survival (CSS), and freedom-from-treatment failure (FFTF) for patients treated with combined modality *versus* single-modality therapy were 95% *versus* 78% (P = .013), 90% *versus* 67% (P = .025) and 95% *versus* 83% (P = .065) respectively. They concluded that PBL patients treated with combined modality have a superior prognosis and significantly better survival rates than single-modality therapy.

Given that most of the cases with excellent statistical outcome come from DLBCL patients, the reputation for good prognosis of PBL is arguable. Lewis et al. (23) retrospectively analyzed 28 adult patients diagnosed with bone lymphoma (21 lesions were diffuse large B cells) and showed that the survival rate of PBL patients was similar to patients with systemic disease. In addition, compared with patients who received chemotherapy or radiotherapy alone, the survival rate of patients who received the combination therapy did not significantly improve, which is opposed to previous studies.

Nagasaki et al. (24) indicated that biological properties of special extranodal lymphomas may be completely different from their nodal siblings. Anaplastic Large Cell Lymphoma (ALCL) is a subtype of peripheral T-cell lymphoma, divided into two major subtypes based on the presence or absence of a protein called “anaplastic lymphoma kinase (ALK)”. Positive ALK resulted from a characteristic cytogenetic abnormality t(2,5)(p23;q35), which is a favorable prognostic feature. Patients affected in nodal sites with ALK-positive lymphoma usually responded well to the CHOP (cyclophosphamide, doxorubicin, vincristine, prednisone) and other similar chemotherapy, and can achieve long-term remission or cure. ALK-negative patients usually relapse and may need more aggressive treatment, including high-dose chemotherapy and a stem cell transplant. Nagasaki et al. (24) summarized that the overall prognosis of these neoplasms is poor, despite the relatively low IPI (average 1.67). Other than nodal ALCL, primary bone ALCL patients with positive ALK did not show a better prognosis. Therefore, they concluded that the clinical behavior and pathogenesis of T cell lymphoma may vary depending on the involved anatomic sites.

Even in the nodal condition, the management of T-cell lymphoma is mainly extrapolated from the treatment of aggressive B-cell lymphomas. T-cell lymphomas possess a poorer prognosis than aggressive B-cell lymphomas. Since the heterogeneity of PTCL-NOS and its poor outcomes, there is no standard care for treatment. CHOP (cyclophosphamide, doxorubicin, vincristine, and prednisone) and CHOP-like regimens are first-line therapy for PTCL patients. Among the nodal cases, half (47%) of the patients who received first-line treatment were identified as refractory, and one-fifth of them relapsed according to the International T-cell Project. The 3-year overall survival rates were 21% for refractory PTCL patients. The 3-year survival of patients receiving salvage bone marrow transplantation was 3 times that of patients not. According to standards of care worldwide, refractory diseases are associated with a higher risk of death and dismal outcome after progression (25).

As a malignancy in bone with a relatively anatomically closed microenvironment, the primary single bone of lymphoma has an extra treatment option. Guzik’s research (26) showed that surgical treatment can significantly improve prognosis if radical resection is possible. In his work, for those patients treated surgically by radical resection and implantation of the prosthesis, there was currently no evidence of generalized disease during the follow-up period. Guzik also claimed surgical treatment should be performed when radical tumor resection is possible, and radiotherapy and chemotherapy should only be employed in palliative treatment when radical resection is incapable. Surgical procedure may play a more significant role in the treatment rather than a merely histologically-confirmed method.

## Conclusion

Not all primary bone lymphomas have a good prognosis. Different types of primary bone lymphoma may have different prognosis. Histology type is definitely an important prognostic factor of PBL. Because different types of primary bone lymphoma may have completely different prognosis. The biological properties of special extranodal lymphomas may be totally different from their nodal siblings.

Now there is no general consensus on the characteristics and treatment strategies of rare PBL cases. The outcomes of radical resection strategies or bone-marrow transplant need further researches and clinical observation. Chemotherapy and radiotherapy may not be the best strategy for all types of PBL. Surgical procedures may play a more significant role in treatment than just a histologically-confirmed method. The adequate management for these rare types of PBL requires further exploration.

## Data Availability Statement

The original contributions presented in the study are included in the article/supplementary material. Further inquiries can be directed to the corresponding author.

## Ethics Statement

Written informed consent was obtained from the patient’s brother for publication of this case report and accompanying images. 

## Author Contributions

SY: managed and followed up on the outcome of the patient and collected associated clinical data and submitted the manuscript. JX: conducted the biopsy surgery which helped confirm the final diagnosis. All authors contributed to the article and approved the submitted version.

## Funding

The Project supported by the Joint Medical Research Program of Shanghai Jing’an District Science and Technology Commission and Health Commission (Grant No. 2020QN07).

## Conflict of Interest

The authors declare that the research was conducted in the absence of any commercial or financial relationships that could be construed as a potential conflict of interest.

## Publisher’s Note

All claims expressed in this article are solely those of the authors and do not necessarily represent those of their affiliated organizations, or those of the publisher, the editors and the reviewers. Any product that may be evaluated in this article, or claim that may be made by its manufacturer, is not guaranteed or endorsed by the publisher.

## Abbreviations

PBL, primary bone lymphoma; PTCL, peripheral T cell lymphoma.
